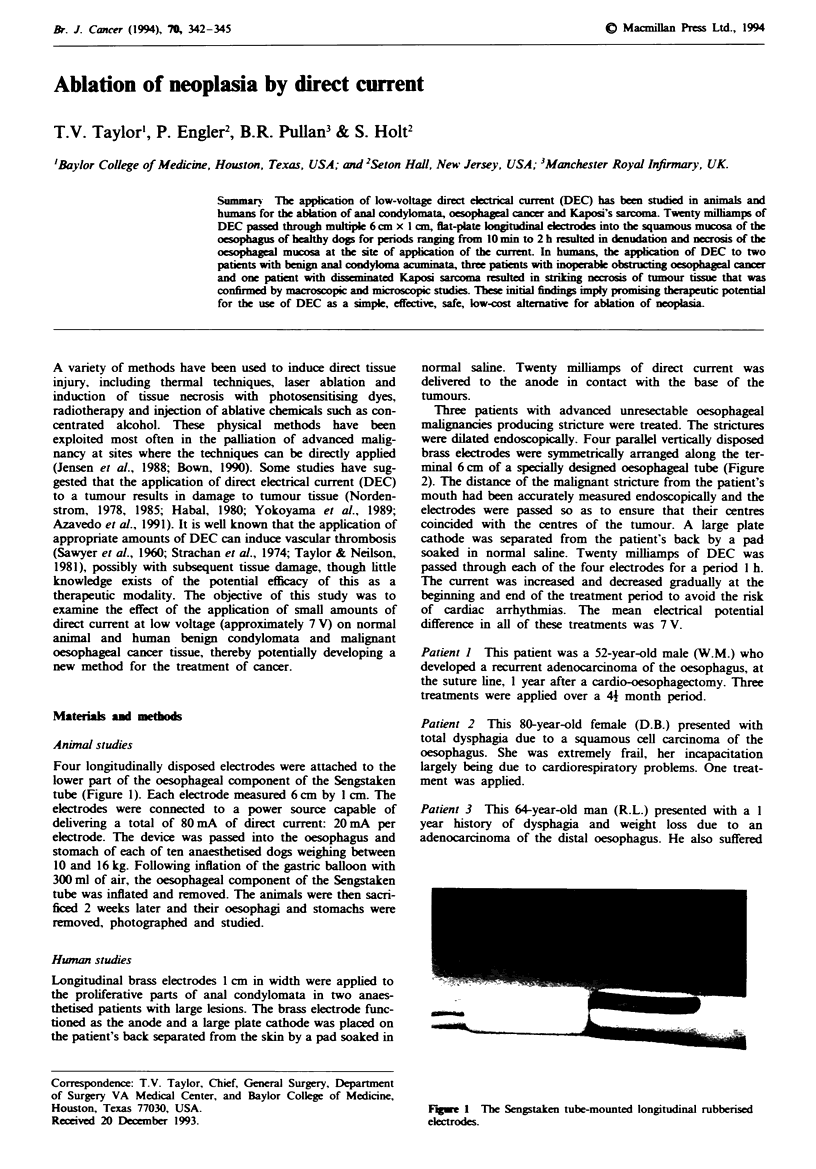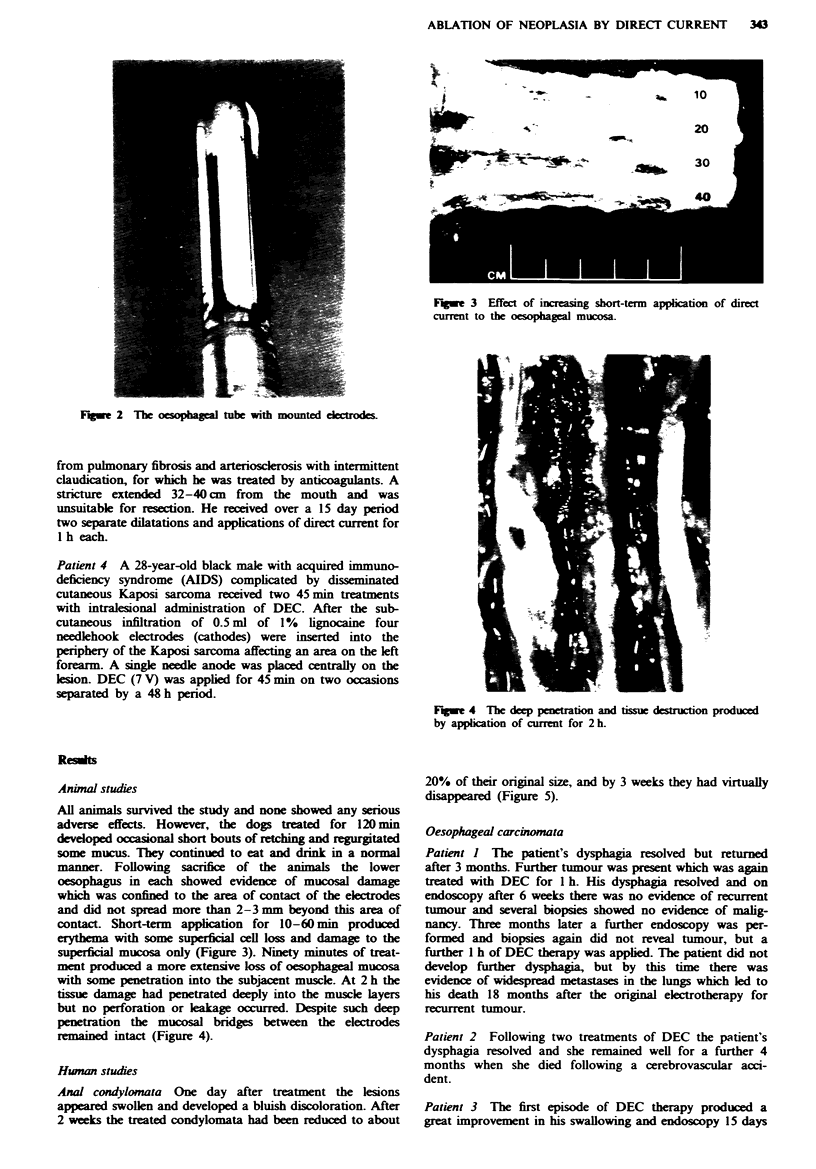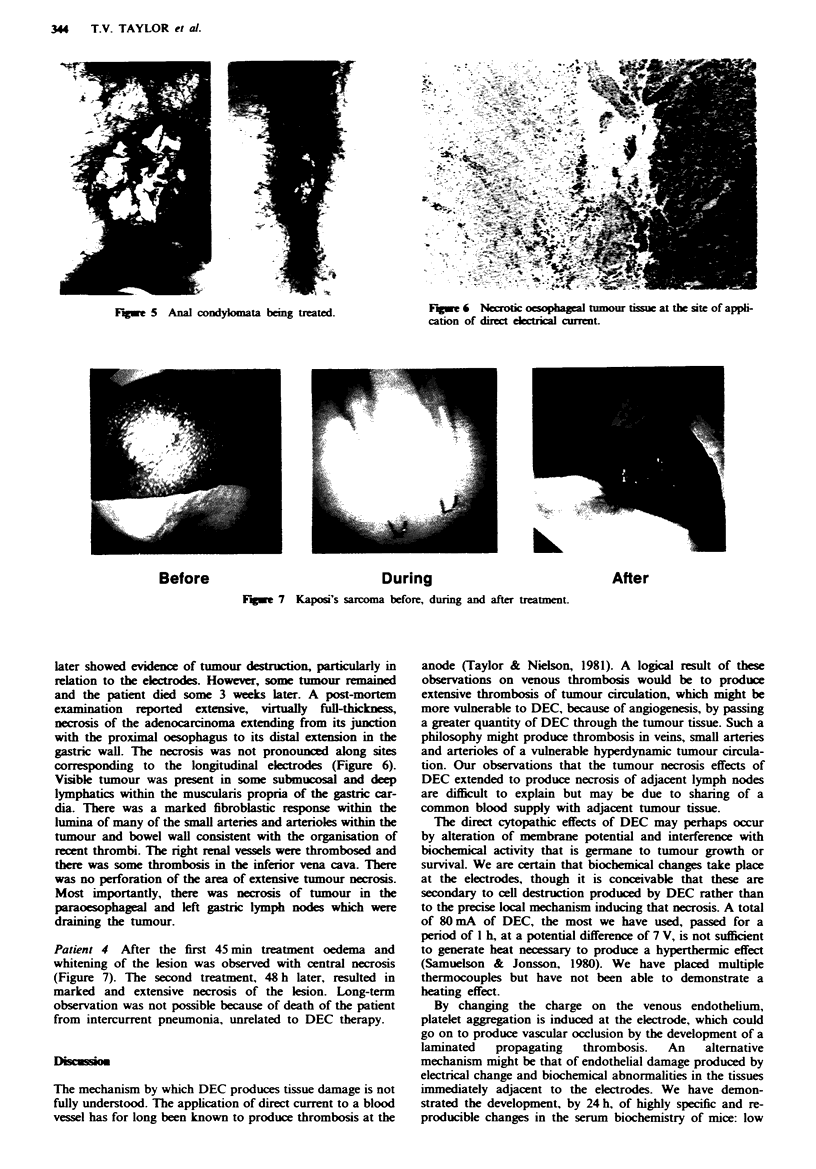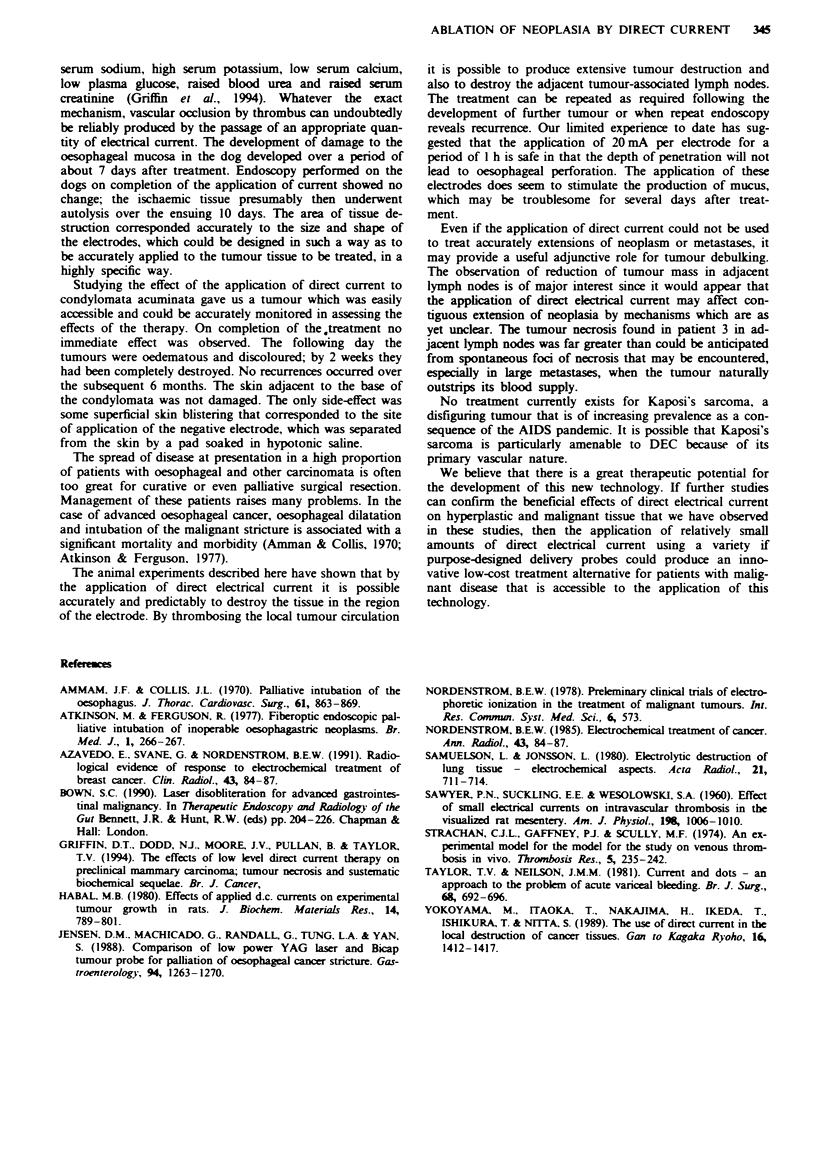# Ablation of neoplasia by direct current.

**DOI:** 10.1038/bjc.1994.304

**Published:** 1994-08

**Authors:** T. V. Taylor, P. Engler, B. R. Pullan, S. Holt

**Affiliations:** Baylor College of Medicine, Houston, Texas.

## Abstract

**Images:**


Br. J. Cancer (1994), 70, 342-345

© Macmillan Press Ltd., 1994

### Ablation of neoplasia by direct current

T.V. Taylor[1], P. Engler[2], B.R. Pullan[3] & S. Holt[2]

[1]Baylor College of Medicine, Houston, Texas, USA; and [2]Seton Hall, New Jersey, USA; [3]Manchester Royal Infirmary, UK.

**Summary** The application of low-voltage direct electrical current (DEC) has been studied in animals and humans for the ablation of anal condylomata, oesophageal cancer and Kaposi's sarcoma. Twenty milliamps of DEC passed through multiple 6 cm × 1 cm, flat-plate longitudinal electrodes into the squamous mucosa of the oesophagus of healthy dogs for periods ranging from 10 min to 2 h resulted in denudation and necrosis of the oesophageal mucosa at the site of application of the current. In humans, the application of DEC to two patients with benign anal condyloma acuminata, three patients with inoperable obstructing oesophageal cancer and one patient with disseminated Kaposi sarcoma resulted in striking necrosis of tumour tissue that was confirmed by macroscopic and microscopic studies. These initial findings imply promising therapeutic potential for the use of DEC as a simple, effective, safe, low-cost alternative for ablation of neoplasia.

A variety of methods have been used to induce direct tissue injury, including thermal techniques, laser ablation and induction of tissue necrosis with photosensitising dyes, radiotherapy and injection of ablative chemicals such as concentrated alcohol. These physical methods have been exploited most often in the palliation of advanced malignancy at sites where the techniques can be directly applied (Jensen *et al.*, 1988; Bown, 1990). Some studies have suggested that the application of direct electrical current (DEC) to a tumour results in damage to tumour tissue (Nordenstrom, 1978, 1985; Habal, 1980; Yokoyama *et al.*, 1989; Azavedo *et al.*, 1991). It is well known that the application of appropriate amounts of DEC can induce vascular thrombosis (Sawyer *et al.*, 1960; Strachan *et al.*, 1974; Taylor & Neilson, 1981), possibly with subsequent tissue damage, though little knowledge exists of the potential efficacy of this as a therapeutic modality. The objective of this study was to examine the effect of the application of small amounts of direct current at low voltage (approximately 7 V) on normal animal and human benign condylomata and malignant oesophageal cancer tissue, thereby potentially developing a new method for the treatment of cancer.

### Materials and methods

### *Animal studies*

Four longitudinally disposed electrodes were attached to the lower part of the oesophageal component of the Sengstaken tube (Figure 1). Each electrode measured 6 cm by 1 cm. The electrodes were connected to a power source capable of delivering a total of 80 mA of direct current: 20 mA per electrode. The device was passed into the oesophagus and stomach of each of ten anaesthetised dogs weighing between 10 and 16 kg. Following inflation of the gastric balloon with 300 ml of air, the oesophageal component of the Sengstaken tube was inflated and removed. The animals were then sacrificed 2 weeks later and their oesophagi and stomachs were removed, photographed and studied.

### *Human studies*

Longitudinal brass electrodes 1 cm in width were applied to the proliferative parts of anal condylomata in two anaesthetised patients with large lesions. The brass electrode functioned as the anode and a large plate cathode was placed on the patient's back separated from the skin by a pad soaked in normal saline. Twenty milliamps of direct current was delivered to the anode in contact with the base of the tumours.

Three patients with advanced unresectable oesophageal malignancies producing stricture were treated. The strictures were dilated endoscopically. Four parallel vertically disposed brass electrodes were symmetrically arranged along the terminal 6 cm of a specially designed oesophageal tube (Figure 2). The distance of the malignant stricture from the patient's mouth had been accurately measured endoscopically and the electrodes were passed so as to ensure that their centres coincided with the centres of the tumour. A large plate cathode was separated from the patient's back by a pad soaked in normal saline. Twenty milliamps of DEC was passed through each of the four electrodes for a period 1 h. The current was increased and decreased gradually at the beginning and end of the treatment period to avoid the risk of cardiac arrhythmias. The mean electrical potential difference in all of these treatments was 7 V.

*Patient 1* This patient was a 52-year-old male (W.M.) who developed a recurrent adenocarcinoma of the oesophagus, at the suture line, 1 year after a cardio-oesophagectomy. Three treatments were applied over a 4½ month period.

*Patient 2* This 80-year-old female (D.B.) presented with total dysphagia due to a squamous cell carcinoma of the oesophagus. She was extremely frail, her incapacitation largely being due to cardiorespiratory problems. One treatment was applied.

*Patient 3* This 64-year-old man (R.L.) presented with a 1 year history of dysphagia and weight loss due to an adenocarcinoma of the distal oesophagus. He also suffered

**Figure 1** The Sengstaken tube-mounted longitudinal rubberised electrodes.

Correspondence: T.V. Taylor, Chief, General Surgery, Department of Surgery VA Medical Center, and Baylor College of Medicine, Houston, Texas 77030, USA.
Received 20 December 1993.

Figure 2 The oesophageal tube with mounted electrodes.

from pulmonary fibrosis and arteriosclerosis with intermittent claudication, for which he was treated by anticoagulants. A stricture extended 32–40 cm from the mouth and was unsuitable for resection. He received over a 15 day period two separate dilatations and applications of direct current for 1 h each.

*Patient 4* A 28-year-old black male with acquired immunodeficiency syndrome (AIDS) complicated by disseminated cutaneous Kaposi sarcoma received two 45 min treatments with intralesional administration of DEC. After the subcutaneous infiltration of 0.5 ml of 1% lignocaine four needlehook electrodes (cathodes) were inserted into the periphery of the Kaposi sarcoma affecting an area on the left forearm. A single needle anode was placed centrally on the lesion. DEC (7 V) was applied for 45 min on two occasions separated by a 48 h period.

### Results

### *Animal studies*

All animals survived the study and none showed any serious adverse effects. However, the dogs treated for 120 min developed occasional short bouts of retching and regurgitated some mucus. They continued to eat and drink in a normal manner. Following sacrifice of the animals the lower oesophagus in each showed evidence of mucosal damage which was confined to the area of contact of the electrodes and did not spread more than 2–3 mm beyond this area of contact. Short-term application for 10–60 min produced erythema with some superficial cell loss and damage to the superficial mucosa only (Figure 3). Ninety minutes of treatment produced a more extensive loss of oesophageal mucosa with some penetration into the subjacent muscle. At 2 h the tissue damage had penetrated deeply into the muscle layers but no perforation or leakage occurred. Despite such deep penetration the mucosal bridges between the electrodes remained intact (Figure 4).

### *Human studies*

*Anal condylomata* One day after treatment the lesions appeared swollen and developed a bluish discoloration. After 2 weeks the treated condylomata had been reduced to about

Figure 3 Effect of increasing short-term application of direct current to the oesophageal mucosa.

Figure 4 The deep penetration and tissue destruction produced by application of current for 2 h.

20% of their original size, and by 3 weeks they had virtually disappeared (Figure 5).

### *Oesophageal carcinomata*

*Patient 1* The patient's dysphagia resolved but returned after 3 months. Further tumour was present which was again treated with DEC for 1 h. His dysphagia resolved and on endoscopy after 6 weeks there was no evidence of recurrent tumour and several biopsies showed no evidence of malignancy. Three months later a further endoscopy was performed and biopsies again did not reveal tumour, but a further 1 h of DEC therapy was applied. The patient did not develop further dysphagia, but by this time there was evidence of widespread metastases in the lungs which led to his death 18 months after the original electrotherapy for recurrent tumour.

*Patient 2* Following two treatments of DEC the patient's dysphagia resolved and she remained well for a further 4 months when she died following a cerebrovascular accident.

*Patient 3* The first episode of DEC therapy produced a great improvement in his swallowing and endoscopy 15 days

**Figure 5** Anal condylomata being treated.

**Figure 6** Necrotic oesophageal tumour tissue at the site of application of direct electrical current.

**Figure 7** Kaposi's sarcoma before, during and after treatment.

later showed evidence of tumour destruction, particularly in relation to the electrodes. However, some tumour remained and the patient died some 3 weeks later. A post-mortem examination reported extensive, virtually full-thickness, necrosis of the adenocarcinoma extending from its junction with the proximal oesophagus to its distal extension in the gastric wall. The necrosis was not pronounced along sites corresponding to the longitudinal electrodes (Figure 6). Visible tumour was present in some submucosal and deep lymphatics within the muscularis propria of the gastric cardia. There was a marked fibroblastic response within the lumina of many of the small arteries and arterioles within the tumour and bowel wall consistent with the organisation of recent thrombi. The right renal vessels were thrombosed and there was some thrombosis in the inferior vena cava. There was no perforation of the area of extensive tumour necrosis. Most importantly, there was necrosis of tumour in the paraoesophageal and left gastric lymph nodes which were draining the tumour.

*Patient 4* After the first 45 min treatment oedema and whitening of the lesion was observed with central necrosis (Figure 7). The second treatment, 48 h later, resulted in marked and extensive necrosis of the lesion. Long-term observation was not possible because of death of the patient from intercurrent pneumonia, unrelated to DEC therapy.

### Discussion

The mechanism by which DEC produces tissue damage is not fully understood. The application of direct current to a blood vessel has for long been known to produce thrombosis at the anode (Taylor & Nielson, 1981). A logical result of these observations on venous thrombosis would be to produce extensive thrombosis of tumour circulation, which might be more vulnerable to DEC, because of angiogenesis, by passing a greater quantity of DEC through the tumour tissue. Such a philosophy might produce thrombosis in veins, small arteries and arterioles of a vulnerable hyperdynamic tumour circulation. Our observations that the tumour necrosis effects of DEC extended to produce necrosis of adjacent lymph nodes are difficult to explain but may be due to sharing of a common blood supply with adjacent tumour tissue.

The direct cytopathic effects of DEC may perhaps occur by alteration of membrane potential and interference with biochemical activity that is germane to tumour growth or survival. We are certain that biochemical changes take place at the electrodes, though it is conceivable that these are secondary to cell destruction produced by DEC rather than to the precise local mechanism inducing that necrosis. A total of 80 mA of DEC, the most we have used, passed for a period of 1 h, at a potential difference of 7 V, is not sufficient to generate heat necessary to produce a hyperthermic effect (Samuelson & Jonsson, 1980). We have placed multiple thermocouples but have not been able to demonstrate a heating effect.

By changing the charge on the venous endothelium, platelet aggregation is induced at the electrode, which could go on to produce vascular occlusion by the development of a laminated propagating thrombosis. An alternative mechanism might be that of endothelial damage produced by electrical change and biochemical abnormalities in the tissues immediately adjacent to the electrodes. We have demonstrated the development, by 24 h, of highly specific and reproducible changes in the serum biochemistry of mice: low serum sodium, high serum potassium, low serum calcium, low plasma glucose, raised blood urea and raised serum creatinine (Griffin *et al.*, 1994). Whatever the exact mechanism, vascular occlusion by thrombus can undoubtedly be reliably produced by the passage of an appropriate quantity of electrical current. The development of damage to the oesophageal mucosa in the dog developed over a period of about 7 days after treatment. Endoscopy performed on the dogs on completion of the application of current showed no change; the ischaemic tissue presumably then underwent autolysis over the ensuing 10 days. The area of tissue destruction corresponded accurately to the size and shape of the electrodes, which could be designed in such a way as to be accurately applied to the tumour tissue to be treated, in a highly specific way.

Studying the effect of the application of direct current to condylomata acuminata gave us a tumour which was easily accessible and could be accurately monitored in assessing the effects of the therapy. On completion of the treatment no immediate effect was observed. The following day the tumours were oedematous and discoloured; by 2 weeks they had been completely destroyed. No recurrences occurred over the subsequent 6 months. The skin adjacent to the base of the condylomata was not damaged. The only side-effect was some superficial skin blistering that corresponded to the site of application of the negative electrode, which was separated from the skin by a pad soaked in hypotonic saline.

The spread of disease at presentation in a high proportion of patients with oesophageal and other carcinomata is often too great for curative or even palliative surgical resection. Management of these patients raises many problems. In the case of advanced oesophageal cancer, oesophageal dilatation and intubation of the malignant stricture is associated with a significant mortality and morbidity (Amman & Collis, 1970; Atkinson & Ferguson, 1977).

The animal experiments described here have shown that by the application of direct electrical current it is possible accurately and predictably to destroy the tissue in the region of the electrode. By thrombosing the local tumour circulation it is possible to produce extensive tumour destruction and also to destroy the adjacent tumour-associated lymph nodes. The treatment can be repeated as required following the development of further tumour or when repeat endoscopy reveals recurrence. Our limited experience to date has suggested that the application of 20 mA per electrode for a period of 1 h is safe in that the depth of penetration will not lead to oesophageal perforation. The application of these electrodes does seem to stimulate the production of mucus, which may be troublesome for several days after treatment.

Even if the application of direct current could not be used to treat accurately extensions of neoplasm or metastases, it may provide a useful adjunctive role for tumour debulking. The observation of reduction of tumour mass in adjacent lymph nodes is of major interest since it would appear that the application of direct electrical current may affect contiguous extension of neoplasia by mechanisms which are as yet unclear. The tumour necrosis found in patient 3 in adjacent lymph nodes was far greater than could be anticipated from spontaneous foci of necrosis that may be encountered, especially in large metastases, when the tumour naturally outstrips its blood supply.

No treatment currently exists for Kaposi's sarcoma, a disfiguring tumour that is of increasing prevalence as a consequence of the AIDS pandemic. It is possible that Kaposi's sarcoma is particularly amenable to DEC because of its primary vascular nature.

We believe that there is a great therapeutic potential for the development of this new technology. If further studies can confirm the beneficial effects of direct electrical current on hyperplastic and malignant tissue that we have observed in these studies, then the application of relatively small amounts of direct electrical current using a variety if purpose-designed delivery probes could produce an innovative low-cost treatment alternative for patients with malignant disease that is accessible to the application of this technology.

### References

AMMAM, J.F. & COLLIS, J.L. (1970). Palliative intubation of the oesophagus. *J. Thorac. Cardiovasc. Surg.*, **61**, 863–869.

ATKINSON, M. & FERGUSON, R. (1977). Fiberoptic endoscopic palliative intubation of inoperable oesophagastric neoplasms. *Br. Med. J.*, **1**, 266–267.

AZAVEDO, E., SVANE, G. & NORDENSTROM, B.E.W. (1991). Radiological evidence of response to electrochemical treatment of breast cancer. *Clin. Radiol.*, **43**, 84–87.

BOWN, S.C. (1990). Laser disobliteration for advanced gastrointestinal malignancy. In *Therapeutic Endoscopy and Radiology of the Gut* Bennett, J.R. & Hunt, R.W. (eds) pp. 204–226. Chapman & Hall: London.

GRIFFIN, D.T., DODD, N.J., MOORE, J.V., PULLAN, B. & TAYLOR, T.V. (1994). The effects of low level direct current therapy on preclinical mammary carcinoma; tumour necrosis and sustematic biochemical sequelae. *Br. J. Cancer*,

HABAL, M.B. (1980). Effects of applied d.c. currents on experimental tumour growth in rats. *J. Biochem. Materials Res.*, **14**, 789–801.

JENSEN, D.M., MACHICADO, G., RANDALL, G., TUNG, L.A. & YAN, S. (1988). Comparison of low power YAG laser and Bicap tumour probe for palliation of oesophageal cancer stricture. *Gastroenterology*, **94**, 1263–1270.

NORDENSTROM, B.E.W. (1978). Preleminary clinical trials of electrophoretic ionization in the treatment of malignant tumours. *Int. Res. Commun. Syst. Med. Sci.*, **6**, 573.

NORDENSTROM, B.E.W. (1985). Electrochemical treatment of cancer. *Ann. Radiol.*, **43**, 84–87.

SAMUELSON, L. & JONSSON, L. (1980). Electrolytic destruction of lung tissue – electrochemical aspects. *Acta Radiol.*, **21**, 711–714.

SAWYER, P.N., SUCKLING, E.E. & WESOLOWSKI, S.A. (1960). Effect of small electrical currents on intravascular thrombosis in the visualized rat mesentery. *Am. J. Physiol.*, **198**, 1006–1010.

STRACHAN, C.J.L., GAFFNEY, P.J. & SCULLY, M.F. (1974). An experimental model for the model for the study on venous thrombosis in vivo. *Thrombosis Res.*, **5**, 235–242.

TAYLOR, T.V. & NEILSON, J.M.M. (1981). Current and dots – an approach to the problem of acute variceal bleeding. *Br. J. Surg.*, **68**, 692–696.

YOKOYAMA, M., ITAOKA, T., NAKAJIMA, H., IKEDA, T., ISHIKURA, T. & NITTA, S. (1989). The use of direct current in the local destruction of cancer tissues. *Gan to Kagaka Ryoho*, **16**, 1412–1417.